# Delay in seeking medical care among breast cancer patients with malignant fungating wounds and their caregivers: a qualitative dyadic study

**DOI:** 10.3389/fpubh.2026.1858757

**Published:** 2026-07-20

**Authors:** Huihui Zhao, Wenji Li, Yanni Wu, Hui Yang, Chunlan Zhou

**Affiliations:** 1 Nanfang Hospital, Southern Medical University, Guangzhou, China; 2 School of Nursing, Southern Medical University, Guangzhou, China

**Keywords:** breast cancer, dyadic, family caregiver, malignant fungating wounds, patient delay

## Abstract

**Background:**

Malignant fungating wounds in breast cancer cause distressing physiological symptoms and profound psychological burdens. Despite the severe and visible nature of malignant fungating wounds, paradoxical care-seeking delays persist, severely impacting patients’ quality of life and survival. However, the exact mechanisms underlying this delaying behavior, particularly from a patient-caregiver interactive perspective within a Chinese cultural context remain unexplored. This study aimed to explore the dyadic experiences and decision-making mechanisms of care-seeking delay using the Theory of Planned Behavior (TPB).

**Methods:**

Based on the Theory of Planned Behavior, data were collected with face-to-face semi-structured interviews from January 2024 to January 2025. Thematic analysis method was used to analysis the data.

**Results:**

Thirteen patient-caregiver dyads were interviewed. Five major themes, strongly aligned with the TPB framework, emerged: (1) Negative attitude and cognitive deficit; (2) The pathway to delay: dyadic behaviors of concealment and dissuasion; (3) Low perceived behavioral control of patient-caregiver dyads; (4) Crisis-triggered transition of medical-seeking intention; and (5) Unspoken support needs of patient-caregiver dyads.

**Conclusion:**

The intention to delay care in breast cancer patients with malignant fungating wounds is not isolated but is heavily sculpted by internalized stigma and mutual protective behaviors within the family dyad. To mitigate patient delay, clinical interventions should shift from conventional health education to targeted, family-centered psycho-education that addresses wound-related stigma and empowers caregivers in early symptom identification.

## Introduction

Worldwidely, breast cancer remains the most common cancer (1). The progression of this disease can lead to profoundly disruptive physical and psychosocial crises, particularly when malignant fungating wounds develop (2). These wounds, which are the result of cancer cells infiltrating the skin (3), evolve into ulcerating, exophytic lesions that are not only highly visible and profoundly disfiguring but are also associated with persistent, copious exudate, bleeding, and a deeply distressing, pervasive malodor (2). The shocking and unrelenting nature of these symptoms transforms the act of seeking medical care into a deeply challenging endeavor for both patients and their families, potentially leading to critical delays. In China, the incidence rate of breast cancer is increasing, and breast cancer has become the second leading cause of cancer death among females (4). Malignant fungating wounds are a common and devastating complication, with studies reporting that approximately 47.1% of breast cancer patients may present with these wounds at the time of diagnosis (5). Unlike localized or subcutaneous malignant nodules, a malignant fungating wound characteristically evolves into an ulcerating, open, and exophytic lesion that breaches the skin’s integrity (6, 7). Failure to completely hide the wound and its unpleasant odor may arouse feelings of shame and severely impair patients’ physical and mental well-being (8). Pain, reduced mobility, social seclusion and emotional distress frequently trouble patients. Breast cancer patients with malignant wounds and their family caregivers often experience distressing physiological and psychosocial effects (5).

With the proposal of the concept of proactive health, despite early detection and treatment of cancer are strongly advocated, cancer delay remains major challenge for global healthcare systems (9). The clinical delay is divided into patient delay, professional delay and treatment delay (10). Although patient delay is traditionally defined as a period of ≥ 3 months from the onset of suspicious symptoms to seeking medical consultation (11, 12), it is a complex variable affected by individual discrepancies and multiple influencing factors (13). Patient delay, especially cancer delay, often represent major barrier in timely care (12), which become important concerns for healthcare systems all around the word (9). The literature shows that patient delay is concerned with the functional impairment, poorer outcomes and/or lower survival (14). It has been proven that timely multidisciplinary therapy may contributes to controlling symptom to prevent disease from progression, improving patient’s quality of life and improving overall survival rates (15). How to improve patient delay behavior of breast cancer patients with malignant wound and to treatment disease earlier is an urgent issue that needs to be solved. Better understanding of patient delay is beneficial to provide timely healthcare for breast cancer patients with malignant wound. The theory of planned behavior (TPB) can be used to predict and understand this behavior. TPB is defined as individual’s motivation to perform behavior that is strongly affected by their intentions, which serves as the most proximal determinant of their behavior (16). This theory incorporates multiple dimensions, such as attitudes, social norms, and the perception of control over behavior (17). It has been widely applied in many researches regarding patient delays.

To the best our knowledge, there are several studies have explored the patient delay of breast cancer patients, including the prevalence and determinants of the delays in breast cancer diagnosis and management (14, 18–21). The reasons for patient delay including objective and nonobjective factors, such as lack of awareness and knowledge of breast cancer symptoms, shyness, symptom disclosure to others, appraisal delay, less physical examination and so on (14, 18–21). Beyond patient-related factors, family caregivers also affect patient behavior patients’ healthcare-seeking behavior (22). In many contexts, particularly in familial-centric societies, the decision to seek medical care is not made autonomously by the patient but is a dyadic process, heavily influenced by the caregiver’s perceptions and interpretations. Caregivers often act as gatekeepers to the healthcare system, and their understanding of the wound and its implications can significantly expedite or impede help-seeking behavior. Prior studies in other diseases have investigated several interconnected issues related to how caregiver understanding contributes to delayed presentation. Misattribution of symptoms by caregivers, their emotional and cognitive reactions, and insufficient disease-specific knowledge or poor health literacy are common caregiver-related factors contributing to delayed care-seeking behavior (22–25). This complex interplay between the caregiver’s comprehension and the patient’s condition creates a dyadic delay, where the fears and misperceptions of both individuals reinforce each other, pushing the window for early intervention further out of reach. While previous studies have separately touched upon patient delays and caregiver burden, few studies focused on the delaying behaviors of Chinese breast cancer patients with malignant wound and their family caregivers with culture-specific considerations. The underlying experiences for patient delays behavior in breast cancer patients with malignant wound and their family caregivers have not been thoroughly understood, which may impede the ability for healthcare professionals to provide better support. Therefore, the current study conducted a qualitative method based on TPB to explore the experiences of breast cancer patients with malignant wound and their family caregivers presented late to treatment.

## Methods

### Study design

A descriptive qualitative research method using semi-structured interviews was carried out. Participants were recruited through purposive sampling. The consolidated criteria for reporting qualitative research checklist (COREQ) was applied to report study findings.

### Participants

Participants were recruited from the breast surgery department of a tertiary hospital in in Guangzhou, China, from January 2024 to January 2025. The inclusion of patients was (1) patients diagnosed with breast cancer malignant wound. (2) Seeking hospital-based treatment at least 3 months after the first onset of symptoms. (3) voluntary participation with informed written consent. The inclusion of caregivers was (1) lived with breast cancer patients, (2) aged 18 years or older, (3) voluntary participation with informed written consent. Patients and caregivers were excluded if (1) suffering from mental illness. (2) Inability to communicate. Finally, a total of 13 patient-caregiver dyads participated in the study.

### Data collection

Data collection was conducted with face-to-face semi-structured interviews. Both patients and caregivers were interviewed separately. A series of questions based on TPB was utilized during the interview (Table 1). We pre-tested two interviews who were not included in this study during the pilot phase. Interviews were conducted by the first author, who is a skilled qualitative female interviewer. She is a nursing PhD student who specializing in systematic learning related to qualitative research. Throughout the interview, questioning, repetition, and summarization were used by the researchers to encourage patient to express their thoughts freely. The interviews lasted 15–40 min. Each interview was audio-recorded for transcription with a mobile phone. Transcripts were returned back to participants for correction. Data collection ceased upon reaching information saturation. Participants’ demographic characteristics (e.g., age, degree of education) and health-related information (e.g., treatment therapy) were also collected. Interviews were conducted in a quiet and private room in the researchers’ workplace.

**Table 1:** Outline of the interview.

Outline of the interview
For Patients: 1. Prior to your current symptoms, did you have any knowledge about breast cancer symptoms? How often do you undergo breast physical examinations? 2. Could you specify the exact time when you first noticed any symptoms in your breast? Were any of these symptoms related to malignant fungating wounds? 3. How did you feel immediately after noticing the symptoms? What actions did you take in response to these symptoms? Please elaborate on the reasons for taking these actions and how you obtained the relevant information (e.g., from medical professionals, family, or online resources). 4. Could you describe the timeline from when you first noticed symptoms to your first hospital visit? Why did you delay seeking medical care? Were there any specific events or factors that eventually prompted you to seek medical attention? 5. At what point did your family caregivers become aware of your symptoms? What actions did they take after learning about your condition, and how did these actions influence your decision to seek medical care? 6. What kind of support do you hope to receive? For family caregivers: 1. When you first learned about the patient’s breast cancer symptoms (including any malignant fungating wounds), what was your initial perception of the severity of the condition? Did you recognize the need for immediate medical care at that time? 2. Could you describe the process of the patient’s delay in seeking medical care? What actions did you take during this period? How did these actions affect the patient’s decision-making? 3. What factors do you think contributed to the delay in seeking medical care? 4. Did you provide any care for the patient’s malignant fungating wounds during the delay period (e.g., cleaning, dressing, or managing symptoms)? 5. What kind of support do you hope to receive?

### Data analysis

Reflexive thematic analysis was performed following the six-step framework proposed by Braun and Clarke (26, 27), with the assistance of QSR NVivo 12.0 software. An inductive approach was adopted for data organization and analysis. The standard six-step analytical procedure includes: (1) data familiarization through repeated reading of interview transcripts and documentation of initial analytical thoughts; (2) systematic generation of preliminary codes; (3) identification of potential themes by collating and grouping relevant codes; (4) theme review to verify the internal coherence, rationality and distinctiveness of all preliminarily identified themes; (5) definition and labelling of themes to accurately capture their core connotations; and (6) compilation of the final analytical report.

The recording audio was transcribed within 24 h after each interview by the first author. Numbers (e.g., Patient 1) were used to replace participants’ names to ensure confidentiality. Two researchers read interview transcripts independently and carefully. One researcher performed initial transcript coding and developed the preliminary codebook, while the other collated and categorized coded data to generate preliminary themes. To preserve the dyadic nature of the patient–caregiver relationship, each patient–caregiver dyad was treated as a single unit of analysis. A custom dyadic matrix was constructed, with each row representing an individual dyad and adjacent columns systematically presenting codes extracted from patients and their corresponding caregivers on core research topics. This structure enabled a rigorous examination of intra-dyadic convergence and divergence in shared experiences, allowing the research team to document congruent, complementary, and contradictory accounts within each dyad. Initial themes derived from intra-dyadic comparisons were further refined via inter-dyadic analysis, which identified consistent and divergent patterns across different dyads. Throughout the analytical process, iterative cross-checks between original individual transcripts and the dyadic matrix were conducted continuously to ensure that the relational and co-constructed characteristics of dyadic experiences were fully retained in the final thematic outcomes.

The coding team comprised the first author (a clinical nurse) and an associate professor with specialized expertise in breast cancer care and qualitative research methodologies. The team regularly reviewed and revised the codebook through weekly group discussions until full consensus was achieved. Using established procedures, the corresponding author translated the transcripts, codes, categories, and themes into English. These materials were subsequently back-translated and checked for accuracy by a nursing researcher who had studied in England for over 1 year.

### Rigor of study

To ensure credibility, the initial interview outlines were constructed in light of the theory of TPB and nursing experts were invited to assess the interview outlines. Then preliminary interviews were implemented to formulate the final interview outlines. All the interviewers had conducted interviews with some experience before. Two researchers checked and coded the interview data independently to ensure the reliability. The transferability was reinforced by the detailed description of study methods, including participant selection criteria, dada collection and data analysis procedures.

### Ethical considerations

All study procedures were approved by the Ethics Committees of Medical Ethics Committee of Nanfang Hospital (No: NFEC-2023-228).

## Results

**Figure 1:**
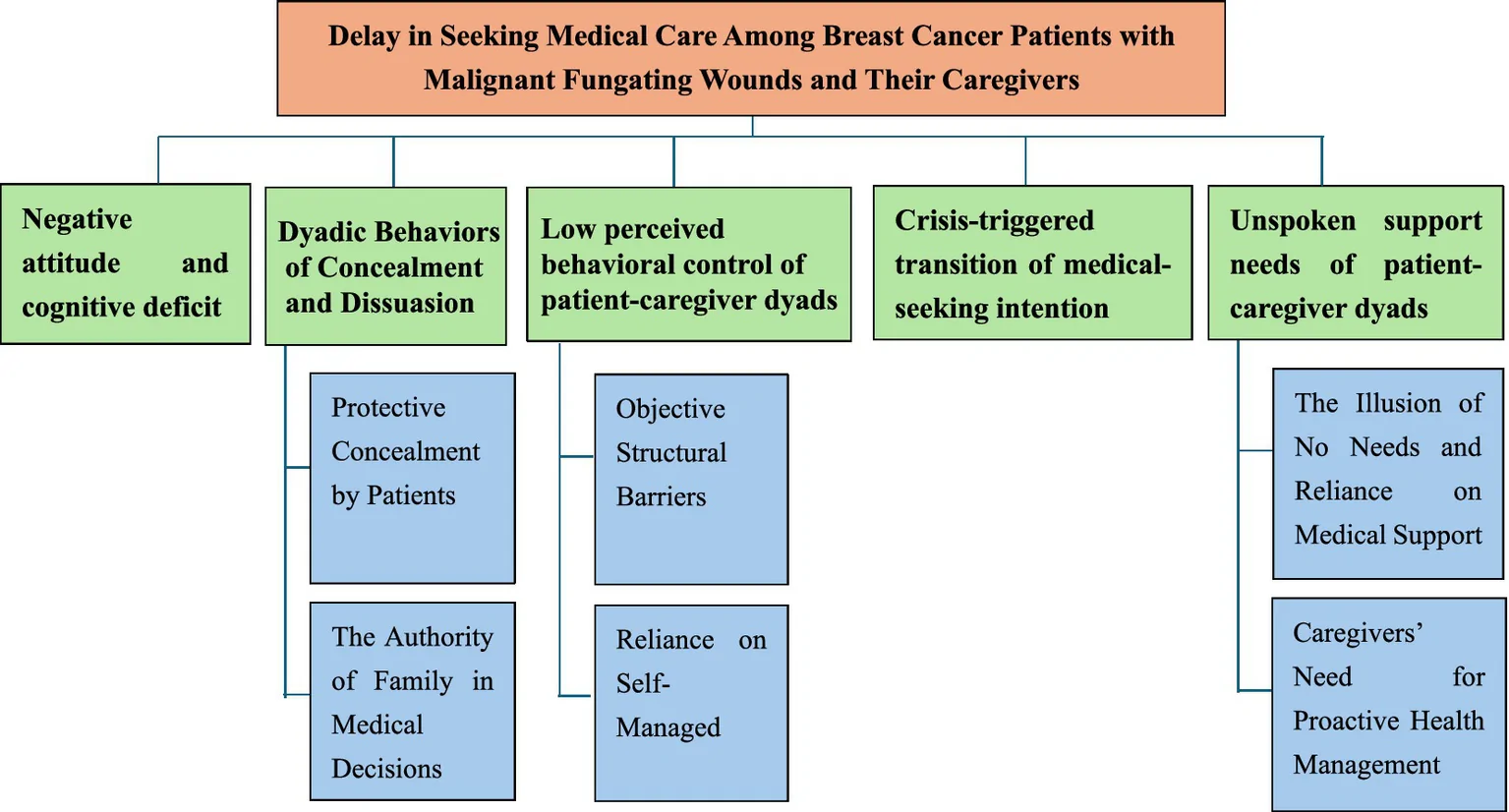
Themes and subthemes of this study.

**Figure 2:**
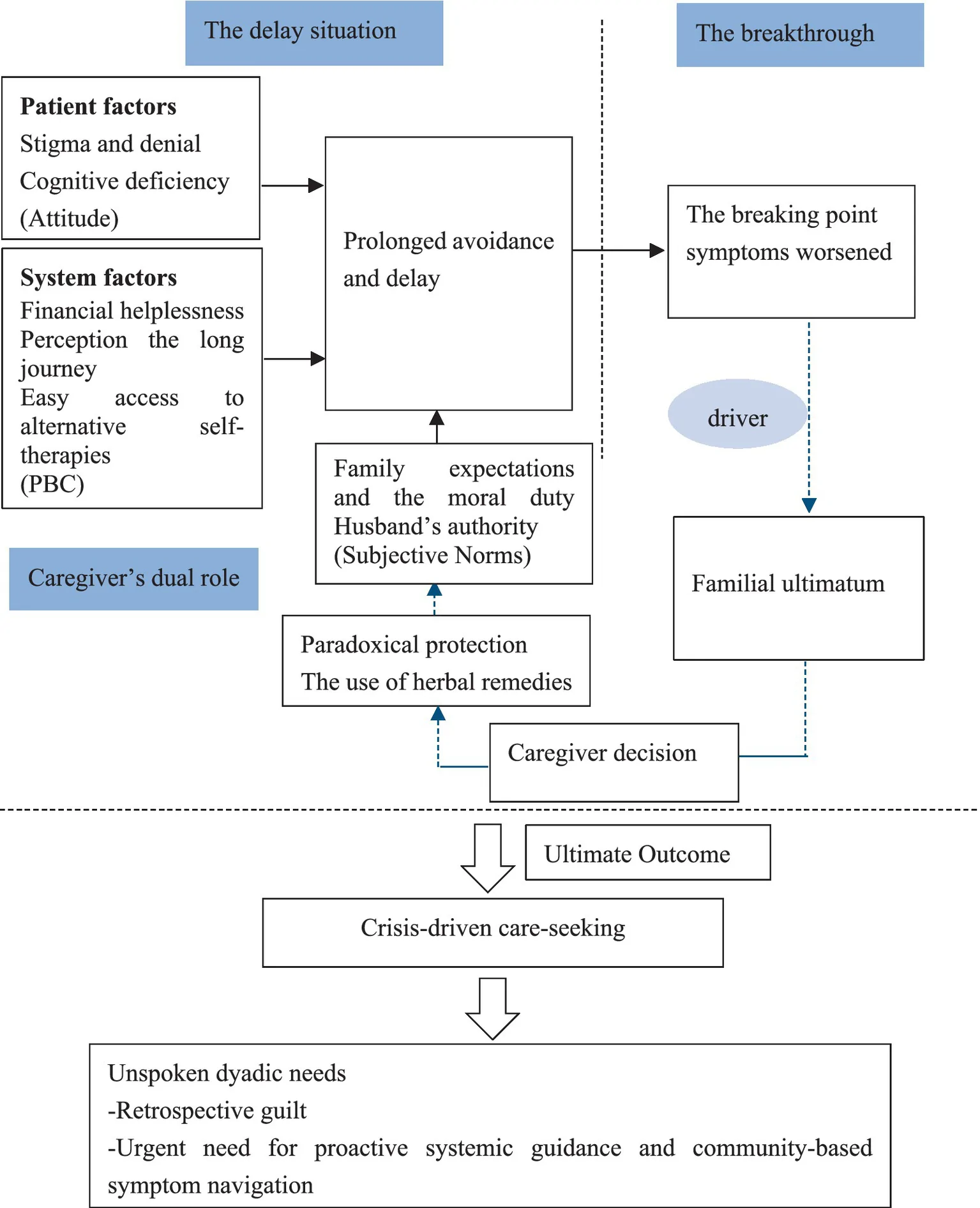
The double-edged sword: a dyadic model of care-seeking delay and breakthrough.

In total, 13 patient-caregiver dyads were included in the data analysis. Table 2 shows the characteristics of the participants. All participants in this study resided in Guangdong Province, and 69.2% were rural residents who received treatment at urban hospitals. Five main themes were identified according to the interviews: (1) Negative attitude and cognitive deficit; (2) Misguided familial support: dyadic protective behaviors inadvertently promote medical care delay; (3) Low perceived behavioral control of patient-caregiver dyads; (4) Crisis-triggered transition of medical-seeking intention; and (5) Unspoken support needs of patient-caregiver dyads. An overview of the thematic framework, illustrating the relationship between the five main themes and their subthemes, is presented in Figure 1. We established a framework of intentions, behaviors, factors of delay in seeking medical care in patients with breast cancer malignant wound (Figure 2).

**Table 2:** Demographic characteristics of participants.

Variable	Patients (*N* = 13)	Caregivers (*N* = 13)
Age (years)	Mean 60.38, SD (9.45), range (52–79)	Mean 47.15, SD (14.14), range (28–76)
≤29	0 (0.0)	2 (15.4)
30–39	0 (0.0)	3 (23.1)
40–49	0 (0.0)	7 (53.8)
50–59	9 (69.2)	1 (7.7)
≥60	4 (30.8)	0 (0.0)
Gender		
Male	0 (0.0)	11 (84.6)
Female	13 (100.0)	2 (15.4)
Occupational status		
Employed	3 (23.1)	9 (69.2)
Unemployed	10 (76.9)	4 (30.8)
Education level		
Primary school	6 (46.2)	1 (7.7)
Middle school	5 (38.5)	5 (38.5)
High school	1 (7.7)	4 (30.8)
College or above	1 (7.7)	3 (23.1)
Marital status		
Married	13 (100.0)	—
Single/Divorced/Widowed	0 (0.0)	—
Medical insurance		
Yes	13 (100.0)	—
No	0 (0.0)	—
Obstetric history		
0	2 (15.4)	—
1	1 (7.7)	—
2	2 (15.4)	—
3	8 (61.5)	—
Residence		
Urban	4 (30.8)	—
Rural	9 (69.2)	—
Patient delay (months)	Mean 7.62, SD (5.89), range (3–24)	—
Caregivers’ relationship with patients		
Spouse	—	7 (53.8)
Daughter/son	—	6 (46.2)

### Theme one: negative attitude and cognitive deficit

This theme captures the dyadic cognitive processes surrounding the malignant wound. It reveals a spectrum of attitudes, not only within the patients themselves but also between patients and their caregivers, ranging from a shared normalization of early symptoms to individualized, deep-seated denial rooted in different existential contexts.

For patients, the delay in seeking help was frequently driven by a profound cognitive deficit in recognizing the wound’s clinical significance. A common pattern was the normalization of the initial skin breach, often attributing it to benign, non-medical causes. This was compounded by ageism, where older adults patients interpreted the deteriorating body as a natural, unworthy-of-investment part of aging. Another distinct pathway was an overestimation of self-efficacy, where patients believed their general problem-solving abilities could be applied to control a symptom. Some patients trivialize their symptoms because they do not understand the manifestations of breast cancer.

*Patient 3: The skin has been broken for about a month, starting after the Loong Boat Festival. I did not know why it (wound) became like this. I did not know it was that serious. I did not know.*

*Patient 2: It has been a long time since this symptom appeared, but I have never cared about it because I am old and do not care about it.*

*Patient 8: I have not told my family (husband and daughter) about my situation.*

*Patient 1: After it (wound) appeared, I still did not pay attention to it. I think it’s because my view of life is different. I can usually help others solve a lot of problems, so I thought I could solve the problem with my breast through my own abilities. I overestimated my capabilities before.*

Caregivers’ dismissal of the wound’s severity was not rooted in personal body neglect, but in a shared illusion with the patient. They co-constructed a benign narrative, attributing the wound to trivial causes. This cognitive alignment, while creating a temporary harmony of inaction, critically delayed medical consultation. A caregiver’s perspective was not about their own body, but a misguided, low-priority assessment of their loved one’s condition.

*Caregiver 5 (Husband): At first, we both thought it was just a little skin infection from the heat. We never linked it to the big disease from before.*

*Caregiver 9 (Child): My mom said it did not hurt, and she looked fine otherwise. I just assumed it was a small ulcer that would heal. I did not push her to see a doctor because she did not seem worried.*

### Theme two: the pathway to delay: dyadic behaviors of concealment and dissuasion

A complex dyadic dynamic emerged where both patients and caregivers inadvertently facilitated delay through misguided protection. These dyadic protective behaviors demonstrate that subjective norms strongly influence delayed care-seeking. Rooted in misguided mutual protection, such behaviors are not isolated individual acts, but manifestations of perceived social expectations, familial roles and cultural values.

#### Protective concealment by patients

Rooted in the fear of burdening family and avoiding conflict, patients engaged in protective concealment behaviors that directly severed communication links essential for timely care. The deeply ingrained cultural script of self-sacrifice, especially for women, led patients to prioritize family harmony over personal health needs. Some patients hide their condition from their family members, who are thus unaware of the patient’s situation. Some patients concealed wounds to avoid becoming a burden on their children.

*Patient 10: All three of my children are still young, and I have not told them about my situation. My husband works far away from home.*

*Patient 3: I do not want to be a burden on my son. The treatment process is just too complicated.*

#### The authority of family in medical decisions

Family plays a critical role in shaping patients’ healthcare-seeking behaviors. Paradoxically, some caregivers contributed to delay by opposing conventional treatment or initiating ineffective home-based remedies. This familial collusion created a social micro-environment where medical consultation was deprioritized to maintain a superficial domestic harmony.

*Patient 5: My husband is very stubborn. He does not want me to go to the hospital for medical treatment and he strongly opposes my receiving chemotherapy. So, I have not come to the hospital for treatment all this time.*

*Patient 6: I do not know what’s wrong with my breast either. My husband treated me with medicine by themself, he said it can help to unclog the mammary glands, and I have not gone to the hospital for a visit.*

### Theme three: low perceived behavioral control of patient-caregiver dyads

The low perceived behavioral control within the dyads was shaped by an interplay of objective structural constraints and subjective perception barriers.

#### Objective structural barriers

From the patients’ perspective, the double burden of limited access to medical technology and financial fragility was a primary source of powerlessness, directly impeding their ability to seek care. Some patients did not have enough money to receive the treatment. Some patients had little access to effective treatment, because of living in rural areas where the medical technology level is limited. They felt trapped by a lack of resources and geographic isolation.

*Patient 9: It will cost a large of money to treat the it (illness), and I do not have that much money for medical treatment. I live in the countryside and I seldom go for a physical examination.*

*Patient 12: The place where I live is far away from the hospital, and it’s very inconvenient for me to go to the hospital for medical treatment every time.*

In response to this shared hardship, caregivers’ narratives revealed a parallel sense of helplessness. This was often expressed not as their own burden, but through their constrained actions and worry for the patient. Their low perceived control was channeled into managing the situation with whatever means were immediately available to them, often validating or participating in the patient’s avoidance of formal healthcare.

*Caregiver 7 (Husband): Seeing her suffer, and knowing we could not afford the hospital… I felt we had no choice but to try the herbal plaster the neighbors talked about.*

#### Reliance on self-managed

A prominent finding was the dyadic pattern of turning to self-managed, non-invasive remedies as a first-line response. For patients, this behavior was a psychological buffer against the fear of intimidating diagnostic procedures, such as biopsies. They perceived readily available, self-applied treatments as safer, more controllable, and less immediately threatening.

*Patient 7: I’m afraid of the breast puncture biopsy operation. Just thinking about it makes me feel like passing out. So, I evaded it. Comparatively, taking herbal remedies is much easier and cheaper, but it (herbal remedies) did not control the disease, the symptoms get worse, so the folk doctor told me to go to the hospital to see a doctor.*

*Patient 10: When that symptom first appeared, I had no idea what the problem was. I did not expect it to be so serious at that time. I just thought that I could get better by taking some herbal remedies.*

Critically, the caregivers’ responses often directly reinforced this delay. Their own low perceived behavioral control and lack of knowledge led them to not only support but actively provide these alternative treatments, interpreting them as a proactive step of care. This dyadic consensus on a non-medical approach created a closed loop, where professional care was only sought when the frightening progression of an uncontrollable wound overrode their shared coping strategy.

*Caregiver 4 (Husband): When the skin of her breast broke and there was some liquid oozing out, I applied herbal remedies to it for her. It has been getting bigger and bigger. That’s when I realized it was beyond our control.*

### Theme four: crisis-triggered transition of medical-seeking intention

The transition from delay to action was rarely proactive; it was predominantly crisis-driven. Seeking medical care occurred only when the breaking point was reached, either through physical crises (uncontrolled malodor or exudate) or through a shift in dyadic power, such as familial persuasion from adult children (e.g., Patient 13), who eventually overruled the patient avoidant intentions.

From the patients’ standpoint, the decision to finally seek care was rarely autonomous. This happened when the physical symptoms of the malignant wound, especially unmanageable foul odor and wound drainage, surpassed the patient’s personal tolerance and self-care ability. Simultaneously, they experienced an overpowering shift in the dyadic power structure, where the persistent persuasion of family caregivers eventually overrode their own avoidant intentions.

*Patient 2: My wound has not been healing properly and it seems even worse now. I just came to the hospital.*

*Patient 13: It was only under the insistence of my son that I came to see a doctor. My son said that as long as I cooperate with the doctor for the examination, he will take care of the rest.*

*Patient 1: My family members were all unaware of my situation before. They just got to know about it recently. It was based on my family’s advice that I decided to come to the hospital for medical treatment this time.*

*Patient 3: My son took me to the hospital to check out my breast. Initially, I was reluctant to attend. However, it was my son who persistently urged me to do so.*

In contrast, caregivers described this transition as a period of intense emotional turmoil and proactive crisis management. They witnessed the patient’s deteriorating physical condition and profound denial, which generated a sense of urgency and responsibility. Their decision to intervene actively, often by persuading the patient firmly or escorting them straight to hospital, represented their self-initiated response to the patient’s severe malignant wound crisis. This action was motivated not only by concern for the patient’s physical health but also by their own psychological distress over witnessing continuous suffering and feeling helpless.

*Caregiver 10 (Husband): Seeing her wound getting worse day by day, and the smell. I could not bear it anymore. I felt that if we did not go to the hospital immediately, something terrible would happen. I had to make the decision for her, even if she was angry with me.*

*Caregiver 13 (Son): My mother just kept avoiding it, saying she’d apply some herbal medicine. But the condition was obviously severe. I felt anxious and heartbroken watching this. Finally, I just told her, I’ve booked the appointment, we are going tomorrow. I could not just stand by and do nothing.*

### Theme five: unspoken support needs of patient-caregiver dyads

While the study unit is the patient-caregiver dyad, the nature of their unspoken needs existed within distinctly different contexts. For patients, the need was rooted in their lived illness experience, whereas for caregivers, the need manifested as a reactive response to the patient’s deteriorating condition and their own prior healthcare decisions.

#### The illusion of no needs and reliance on medical support

For many patients with malignant fungating wounds, the overwhelming uncertainty surrounding their deteriorating physical condition led them to completely abandon seeking further proactive support. They equated care solely with curative treatment, which masked their underlying need for symptom management. Their expressed lack of needs stemmed from a profound sense of helplessness.

*Patient 10: For my situation, I have no other demands. I uncertain about physical condition. All I hope for is that the doctor can offer me the best treatment plan.*

This quote powerfully illustrates the patient’s perceived helplessness (low Perceived Behavioral Control in TPB), where the desperate reliance on the physician’s best plan overshadows any proactive articulation of psychological or wound-care needs.

#### Caregivers’ need for proactive health management

In contrast to the patients’ passive resignation, caregivers’ responses were profoundly shaped by their observations of the patient’s suffering and their retrospective guilt regarding delayed medical care-seeking. Their unspoken need, therefore, was not an independent demand, but a reactive call for systemic change, namely a desire that future families be spared their experience. When discussing the patient’s malignant wound, they expressed a strong need for proactive health management.

*Caregiver 4 (Husband): Our health awareness is so poor, I really wish someone could have reminded me to take her to see a doctor for a physical examination earlier.*

*Caregiver 3 (Son): She (My mom) was old, I should have cared more about her actively.*

This reflection underscores a critical gap in the rural healthcare system. Caregivers are implicitly pleading for a shift from passive medical services to proactive, community-based health navigation that targets not just the patients, but the family decision-makers as well.

## Discussion

Delayed healthcare-seeking is prevalent, particularly in populations with limited health literacy or systemic access barriers. This study aims to examine the experiences of patient delay among Chinese breast cancer patient with malignant wounds and their family caregiver. Our findings reveal that the mechanism of patient delay in breast cancer patients with malignant fungating wounds is distinct from that in patients with early-stage symptoms. Guided by the TPB, patients’ delayed access to medical care cannot be simply ascribed to personal traits including ignorance, indolence and anxiety. Rather, this phenomenon arises from the interaction of social, psychological, familial and medical system factors. It is not merely an accumulation of isolated barriers, but a complex interplay of internalized stigma, distorted protection, and deeply rooted cultural hurdles.

Consistent with previous study, this study emphasizes that family members, especially husband and children, have a great influence on the patient’s decision-making on medical help-seeking behavior, which can be named double effect. On the one hand, caregivers can remind and/or take patients to seek medical treatment as early as possible, on the contrary, they may prolong a patient delay with their own view, these findings are in alignment with the previous researches (28–30). This study also found that there was lack of family communication between patients and their family caregivers. Communication problems were prevalent in families coping with cancer (31). Direct communication with family plays an essential role in increasing health literacy and promoting health-seeking behaviors (32). Good communication with family members may help patients to disclose their symptoms and get enough encourage and assistance to seek medical treatment. In addition, it can also reduce family caregivers’ psychological guilt with regard to the delayed behavior.

According to the TPB framework, attitude serves as a crucial predictor of behavior. Previous research has also demonstrated that attitudes among cancer patients are a prevalent cause of patient delay (10). A substantial number of patients perceived their breast symptoms as minor and overlooked them (33). Unlike previous studies focusing on early-stage breast lumps where symptoms are often concealed or painless, the malignant fungating wounds in our study presented as highly visible, malodorous, and disfiguring lesions. Paradoxically, rather than prompting immediate medical consultation, these severe symptoms triggered the veil of stigma and denial. In an attempt to mitigate the profound psychological trauma of bodily disfigurement, patients often adopted cognitive avoidance. Their negative behavioral attitudes were deeply rooted in the fear and misunderstanding of the malignant fungating wound itself, as well as the anticipated fear of painful procedures and exposing bodily disfigurement. In contrast, caregivers’ behavioral attitudes centered on a weighing of whether seeking medical care would inflict greater suffering on the patient. Our findings reveal that caregivers can become active or passive co-conspirators in the delay, driven by the prioritization of reducing present suffering.

Based on the TPB, perceived behavioral expectations from significant referents such as family and friends ultimately influence behavioral intention, particularly within the Chinese context. Previous literature often attributes delay merely to a lack of knowledge (34). However, our findings highlight a more nuanced cultural phenomenon. Influenced by traditional Chinese values of extreme frugality and altruistic self-sacrifice within the family unit, many older rural women harbor deep-seated fatalism. Traditional Chinese gender expectations cause women to routinely prioritize family responsibilities over their own well-being (33). In their perception, investing limited family financial resources into treating an incurable and shameful wound is unjustified. Consequently, the delay is not simply derived from ignorance, but represents a deliberate, albeit tragic, form of self-silencing to protect their family’s socioeconomic stability. For caregivers, caregivers’ subjective norms originated from the intra-familial cultural norm of filial piety or fulfilling one’s duty (35). This reveals that when seeking care, caregivers not only carry the patient’s suffering but also bear the psychological burden of moral accountability. This intertwined pressure from internal and external norms placed caregivers in a dilemma between the patient’s own wishes and sociocultural expectations. This dynamic conflict in family care decision-making has been critically overlooked in prior single-perspective studies, and our dyadic design clearly unveils it.

More than half of dyads in this study were rural residents, compared with urban residents, they were more prone to patient delay due to time limitations, long travel distances and financial burdens. Patients perceived behavioral control was dominated by a sense of global loss and powerlessness. The caregiver’s perceived behavioral control was essentially constrained by their role overload and resource depletion as the sole caregiver, which was aligns with literature on the burden of breast cancer family caregiving (36). In this context, we found that some breast cancer patients with malignant wounds conducted self-treatment with traditional Chinese herbal therapies rather than seeking medical help in the hospital, which previous studies rarely mentioned. They took traditional Chinese herbal orally or applied it externally to the wound on their own, following folk prescriptions, which is a relatively accessible approach. Although traditional Chinese herbal medicine has a certain therapeutic effect on breast cancer (37), patients and caregivers are not advised to use it on their own or receive such treatment at unregulated medical institutions, as this may instead worsen the condition.

### Implications for practice and research

The profound physiological and psychological devastation caused by malignant fungating wounds in breast cancer demands a paradigm shift in how healthcare systems approach patient delay. Our findings underscore that addressing this delay requires moving beyond generic breast screening program, toward targeted, dyadic, and culturally sensitive interventions.

First, mitigating wound-related stigma is paramount. Clinical practice must evolve from passive symptom management to proactive psychological screening. Healthcare professionals, particularly community nurses in rural areas, should proactively assess for body image distress and internalized stigma. Developing trauma-informed educational materials that specifically destigmatize the appearance and odor of malignant fungating wounds could empower patients to overcome the veil of denial and seek help before reaching a physical crisis. Furthermore, establishing proactive community-based health navigation systems is urgently needed to address the unarticulated needs of rural dyads who often passively resign to fatalism.

Second, interventions must adopt a family-centered (dyadic) approach. Recognizing that family caregivers act as a double-edged sword, healthcare providers cannot treat the patient in isolation. Future research should focus on developing dyadic communication programs tailored to the Chinese cultural context. These programs should aim to dismantle the paradoxical protection (where patients hide to avoid burdening families, and families delay out of misguided care) by equipping caregivers with the health literacy and emotional tools necessary to initiate timely, open conversations about progressing symptoms, thereby transforming them from obstructor into early facilitators of care.

Lastly, targeted health education is essential to address structural obstacles and public misconceptions regarding traditional Chinese herbal therapies. Community health campaigns must explicitly educate residents on the limitations of unguided, home-based herbal remedies for ulcerative lesions. Healthcare providers should emphasize that self-treatment of malignant wounds often exacerbates the disease trajectory and financial burden. Future quantitative studies could evaluate the efficacy of integrating targeted dyadic psycho-education into routine rural healthcare to systematically reduce the incidence of severe malignant wounds presentations.

### Limitations

This study has several limitations. Firstly, all participants were recruited from an urban tertiary hospital, which may affect the representative or generalizable of the results. Secondly, because of the cultural disparities between the English and Chinese languages, the transcription of the interview data also constitutes a constraint on this research.

## Conclusion

Guided by the TPB, this qualitative study identified reasons and process driving patient delay among breast cancer patients with malignant wounds and their caregivers. The delay is fundamentally a dyadic, avoidant coping strategy rather than an individual cognitive deficit, which is related to the internalized stigma, distorted protection, and cultural hurdles. To effectively reduce delays, healthcare systems should implement proactive, family-centered health navigation to address unarticulated dyadic needs, destigmatize wound care, and empower caregivers to transition from silent colluders to active facilitators of early treatment.

## Data Availability

The original contributions presented in the study are included in the article/supplementary material, further inquiries can be directed to the corresponding author.
